# Supplementary material for: The MuvB complex binds and stabilizes nucleosomes downstream of the transcription start site of cell-cycle dependent genes
Source: Nat Commun. 2022 Jan 26;13:526. doi: 10.1038/s41467-022-28094-1 (PMC8792015; doi:10.1038/s41467-022-28094-1)
Supplement: Supplementary file 1 — Supplementary Information [file 41467_2022_28094_MOESM1_ESM.pdf]

Supplementary Information

The MuvB Complex Binds and Stabilizes Nucleosomes Downstream of the Transcription Start Site of Cell-Cycle Dependent Genes

Anushweta Asthana[1], Parameshwaran Ramanan[1], Alexander Hirschi[1], Keelan Z. Guiley[1], Tilini U. Wijeratne[1], Robert Shelansky[2], Michael J. Doody[2], Haritha Narasimhan[1], Hinrich Boeger[2], Sarvind Tripathi[1], Gerd A. Müller[1]*, Seth M. Rubin[1]*

1) Department of Chemistry and Biochemistry, University of California, Santa Cruz, CA 95064, USA

2) Department of Molecular, Cell, and Developmental Biology, University of California, Santa Cruz, CA 95064, USA

* Corresponding authors: gemuelle@ucsc.edu, srubin@ucsc.edu

Supplementary Table 1: Data collection and Refinement statistics.

Data were collected on a single crystal. Values in parentheses are for the highest resolution shell.

**Data collection**	
Beam line	APS(23IDB)
Resolution Range (Å)	60.0-2.55 (2.69 - 2.55)
Space group	C 1 2 1
Unit cell dimensions: a,b,c (Å), α, β, γ (º)	133.58 77.81 64.56<br>90 114.71 90
Wavelength (Å)	1.03
Total observations	43599 (6521)
Unique reflections	19131 (2805)
Completeness (%)	97.4 (97.8)
R_{merge}	13.5 (52.1)
<I/σ>	7.9 (3.5)
$CC_{1/2}$	0.97 (0.47)
Redundancy (highest shell)	2.3 (2.3)
**Refinement**	
R_{work} %/ R_{free} %	16.1/ 25.0
Number of non-hydrogen atoms	4862
Protein	4720
Water	142
Wilson B-factor ($Å^2$)	32.79
RMSD Bond length (Å)	0.008
RMSD Bond angle (°)	0.94
Ramachandran favored(%)/Ramachandran outliers (%)	96.0/0.0

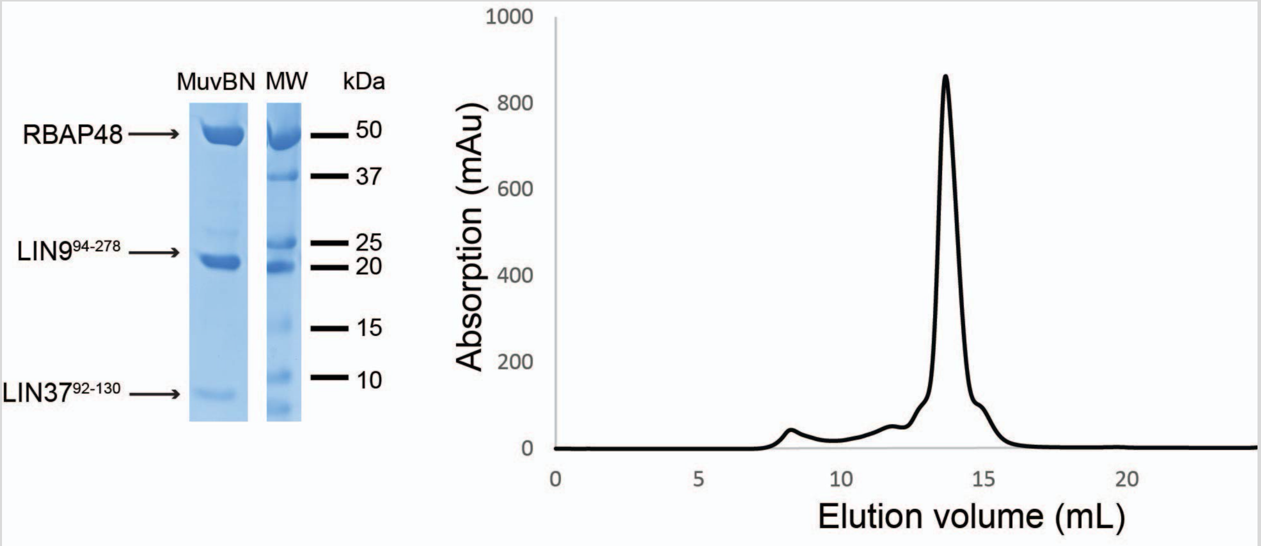

Supplementary Fig. 1: MuvBN reconstitution for crystallization. A stable complex of RBAP48 and the indicated LIN9 and LIN37 constructs eluted on Superdex 200. (Left) Coomassie stained gel of three subunits following purification. (Right) UV (280 nm) absorption trace from size-exclusion chromatography.

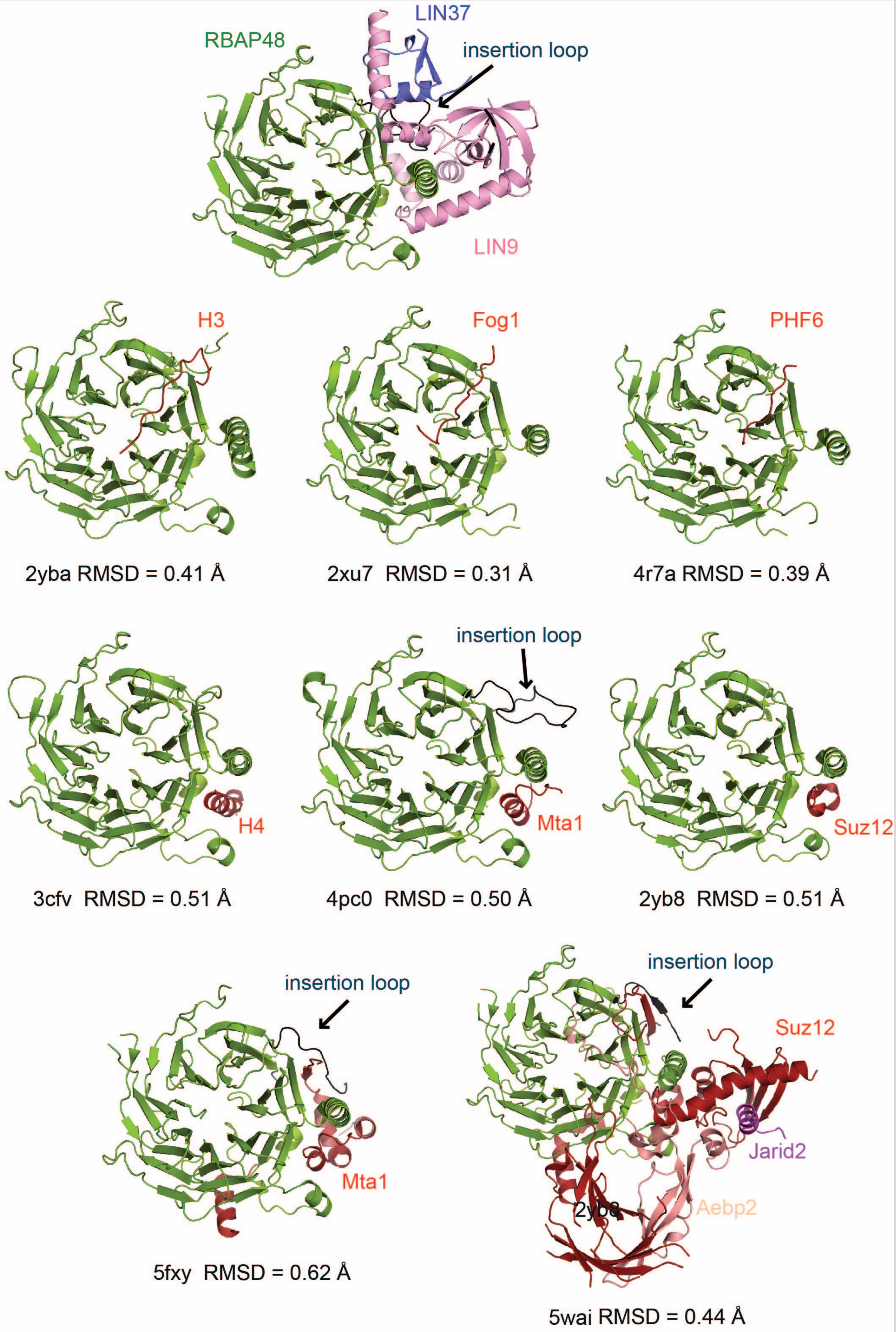

Supplementary Fig. 2: Comparison of RBAP48 structures and interactions with different peptides and proteins. The MuvBN complex structure is shown at the top. The pairwise RMSD for C_α atoms in RBAP48 between each structure and MuvBN is shown. Structures with peptides (second and third rows) show two major binding sites. The H3 binding site across the face of the β-propeller domain (second row) is accessible in MuvBN, while the H4 binding site (third row) is not. The RBAP48 insertion loop (residues 88-115) is not ordered and modeled in nearly all the structures of RBAP48 with small peptides. It appears in the structure with the Mta1 peptide, although in that crystal it is involved in packing. In the structures of larger complexes (bottom row), part of the insertion loop is ordered and forms a strand and packs against an added strand from an RBAP48-interacting partner. In the MuvBN structure, the loop is completely ordered and makes extensive interactions with both LIN9 and LIN37.

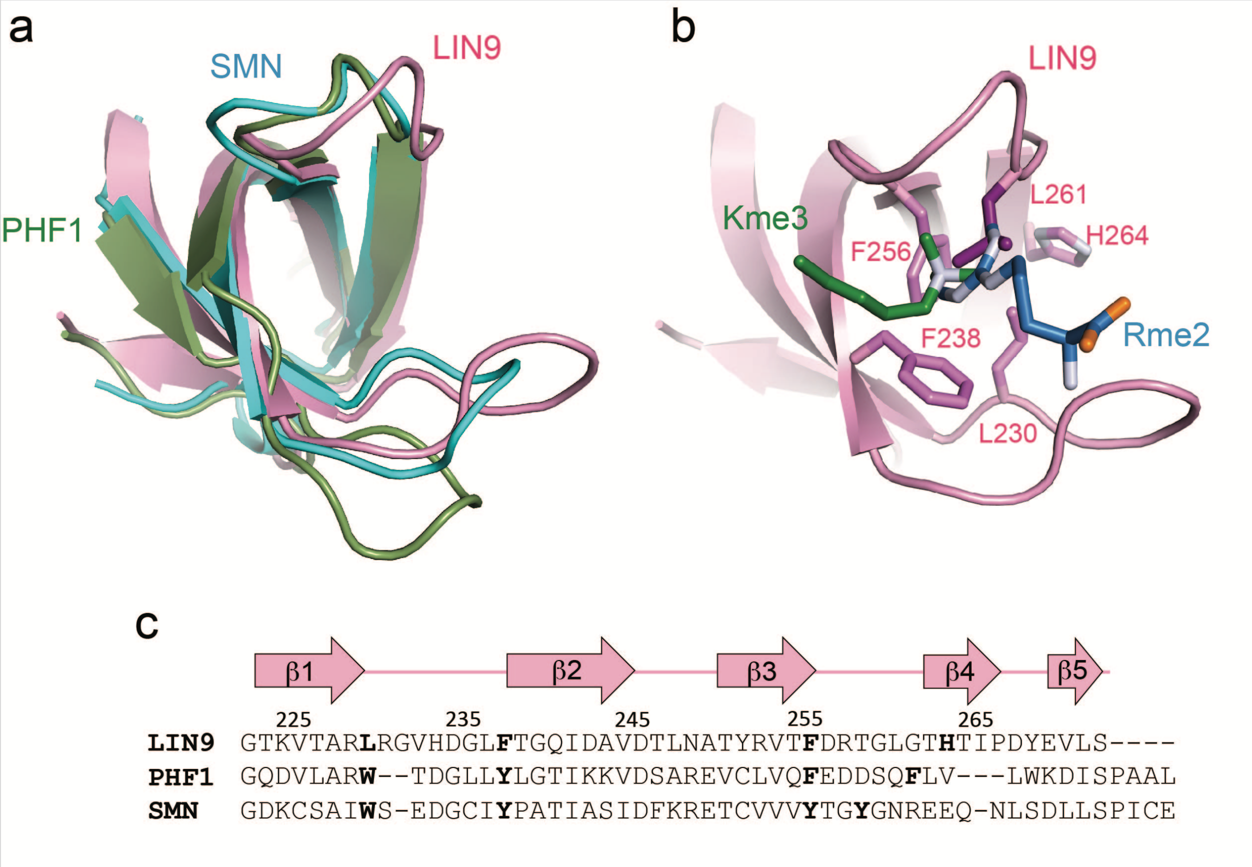

Supplementary Fig. 3: **Structure of the LIN9 Tudor domain and comparison to other Tudor domains.** (**a**) Alignment of the LIN9 Tudor domain with PHF1, which binds a trimethylated peptide in histone 3 (H3K36Me3), and SMN, which recognizes a dimethylated arginine. Pairwise RMSD for C_α atoms are 1.0 Å between LIN9 and PHF1 and 0.9 Å between LIN9 and SMN. (**b**) View of LIN9 residues L230, F238, F256 and H264, which correspond to residues in the other structures that form the aromatic cage. The key modified interacting residue with the PHF1 and SMN structures are modeled in the view. The "cage" in LIN9 contains fewer aromatic residues (alignment shown in (**c**)), and the position of the interacting residue is occupied by L261 in LIN9. We conclude from the structure that the Tudor domain of LIN9 is non-canonical, and it may not interact with modified histone species, or it may interact in a different manner as previously observed.

a

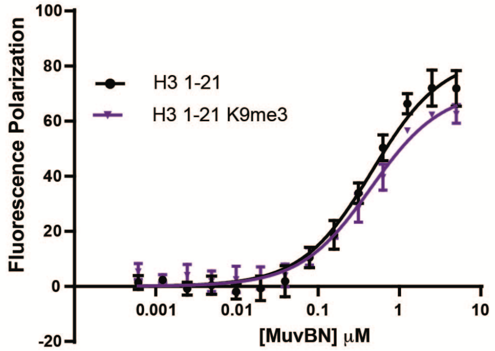

	MuvBN K_d (nM)
H3 1-21	650 ± 110
H3 1-21 K9me3	750 ± 160

c

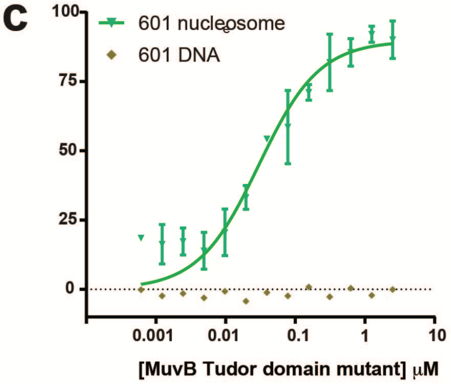

	MuvB Tudor mutant K_d (nM)
601 nucleosome	50 ± 10

b

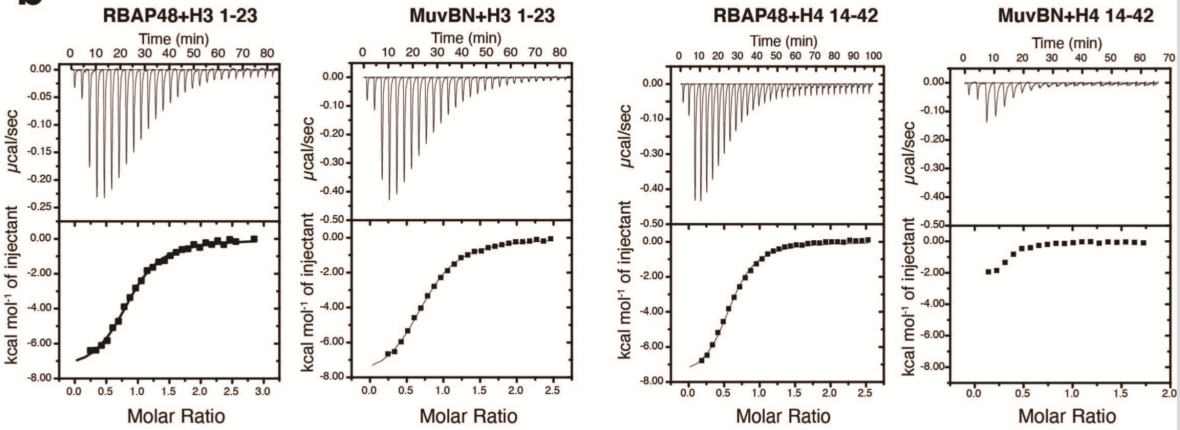

	RBAP48 K_d (nM)	MuvBN K_d (nM)
H3 1-21	950 ± 70	1520 ± 20
H4 14-42	2300 ± 100	no binding detected

Supplementary Fig. 4: Supporting data characterizing MuvB association with nucleosomes. (**a**) Fluorescence polarization (FP) assay as in Fig. 4 but using the MuvBN subcomplex. FP is normalized to dye-labeled probe alone. The data are shown as mean values from three replicates with the standard deviation (SD) as error bars. Data are fit assuming a single binding constant. The affinities and error estimates reported in the table are from global fitting of all data across the three technical replicates. (**b**) Isothermal titration calorimetry measurements of H3 and H4 peptide binding to RBAP48 and MuvB. These data demonstrate MuvB binds H3 similar to RBAP48 alone, and the affinity of H3 for RBAP48 is similar to that previously reported[47]. We suspect that the affinity of MuvBN for H3 is slightly tighter when measured by FP because of the hydrophobic dye on the probe. ITC measurements were made with a Microcal, LLC calorimeter. 1 mM histone was titrated into 50 μM of the indicated protein in a buffer containing 10 mM HEPES pH 7.0, 200 mM NaCl, 5 MM DTT. The data were fit to a one site binding model and the stoichiometry parameter in each case fit to ~1. The reported affinities in the table are the mean values from three replicates with the standard deviation reported as error. (**c**) Fluorescence polarization (FP) assay as in Fig. 4 but using MuvB assembled with the LIN9 Tudor cage mutant (L230A, F238A, F256A, and H264A). FP is normalized to dye-labeled probe alone. The data are shown as mean values from three replicates with the SD as error bars. Data are fit assuming a single binding constant. The affinity and error estimate reported in the table is from global fitting of all data across the three technical replicates. Source FP data are provided as Source Data.

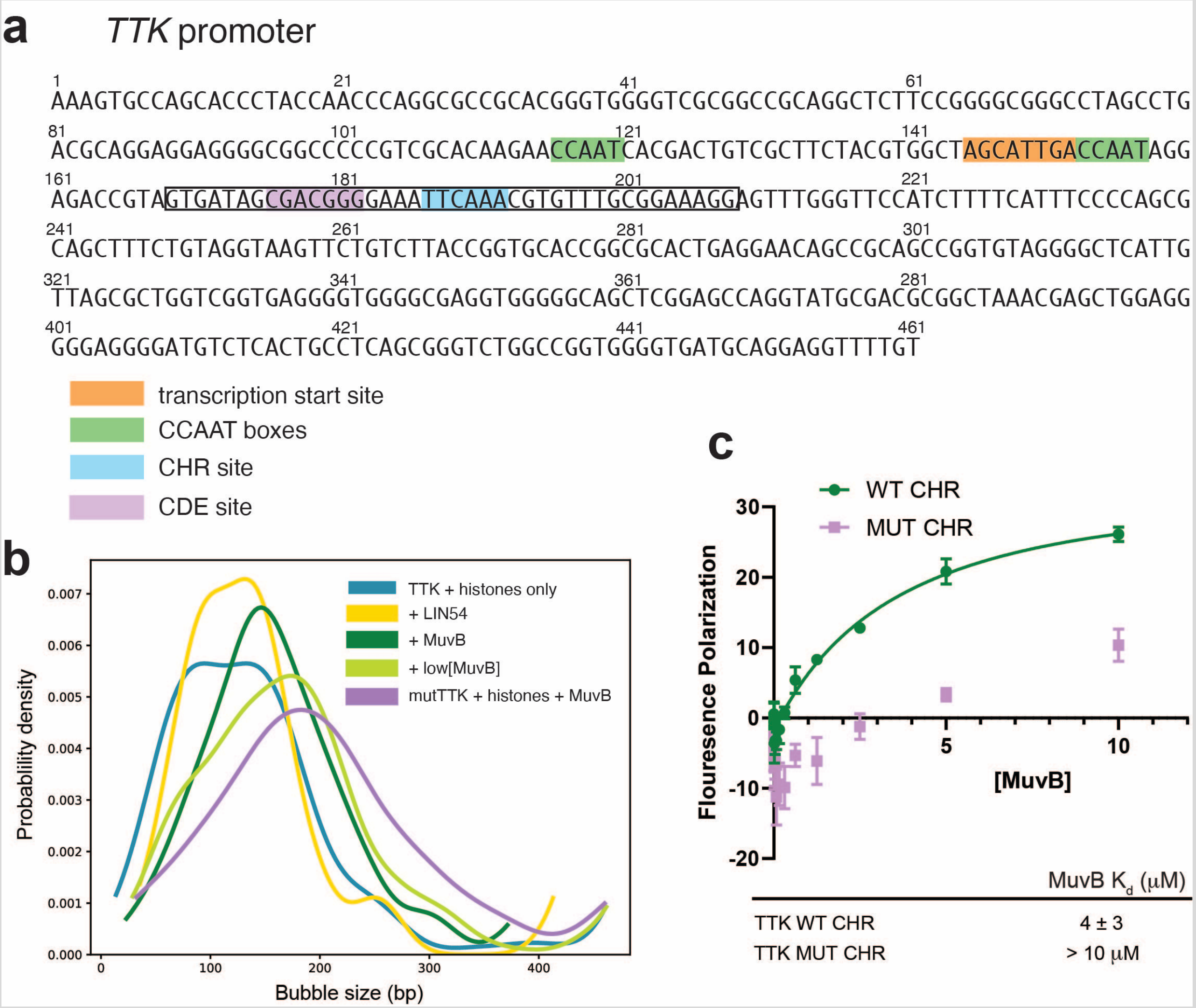

Supplementary Fig. 5: Supporting data for nucleosome crosslinking experiment with reconstituted *TTK* promoter. (**a**) DNA fragment from *TTK* promoter used in these experiments. The boxed sequence corresponds to the fragment used in the binding experiment in panel **c**. (**b**) Estimated probability density functions for bubble size for each of the experimental conditions: blue (*TTK* promoter with histones), gold (+LIN54), dark green (+MuvB), light green (+ low[MuvB]), and purple (mutant *TTK* with histones and MuvB). Considering the resolution of the experiment and allowing for the possibility of partially wrapped octamers, we interpreted bubbles larger than 90 bp as nucleosomes. We have previously shown that a partially unwrapped pre-nucleosomal structure can be distinguished from a fully wrapped nucleosome with this psoralen cross-linking assay[56]. We note that probability density around 90 bp decreases when MuvB but not LIN54 is added to the sample. This shift in the probability density away from pre-nucleosomal to nucleosomal size suggests that MuvB stabilizes the fully wrapped nucleosomes. (**c**) Fluorescence polarization assay measuring the affinity of MuvB for a 40-bp fragment from the TTK promoter (boxed in panel **a**). 20 nM of fluorescein-labeled fragment was titrated with increasing concentrations of purified MuvB in a buffer containing 150mM NaCl, 20mM Tris pH 8.0, and 0.1%Tween-20, and the fluorescence polarization recorded in a 384-well plate (volume 20 μL). The data are shown as mean values from three replicates with the standard deviation (SD) as error bars. Data are fit assuming a single binding constant. The affinities and error estimates reported in the table are from global fitting of all data across the three replicates. The K_d value measured here is similar to that previously reported for LIN54 binding to a similar CHR motif[31]. The scrambled mutTTK is the same sequence but with the CHR sequence relaced with TGCATA.

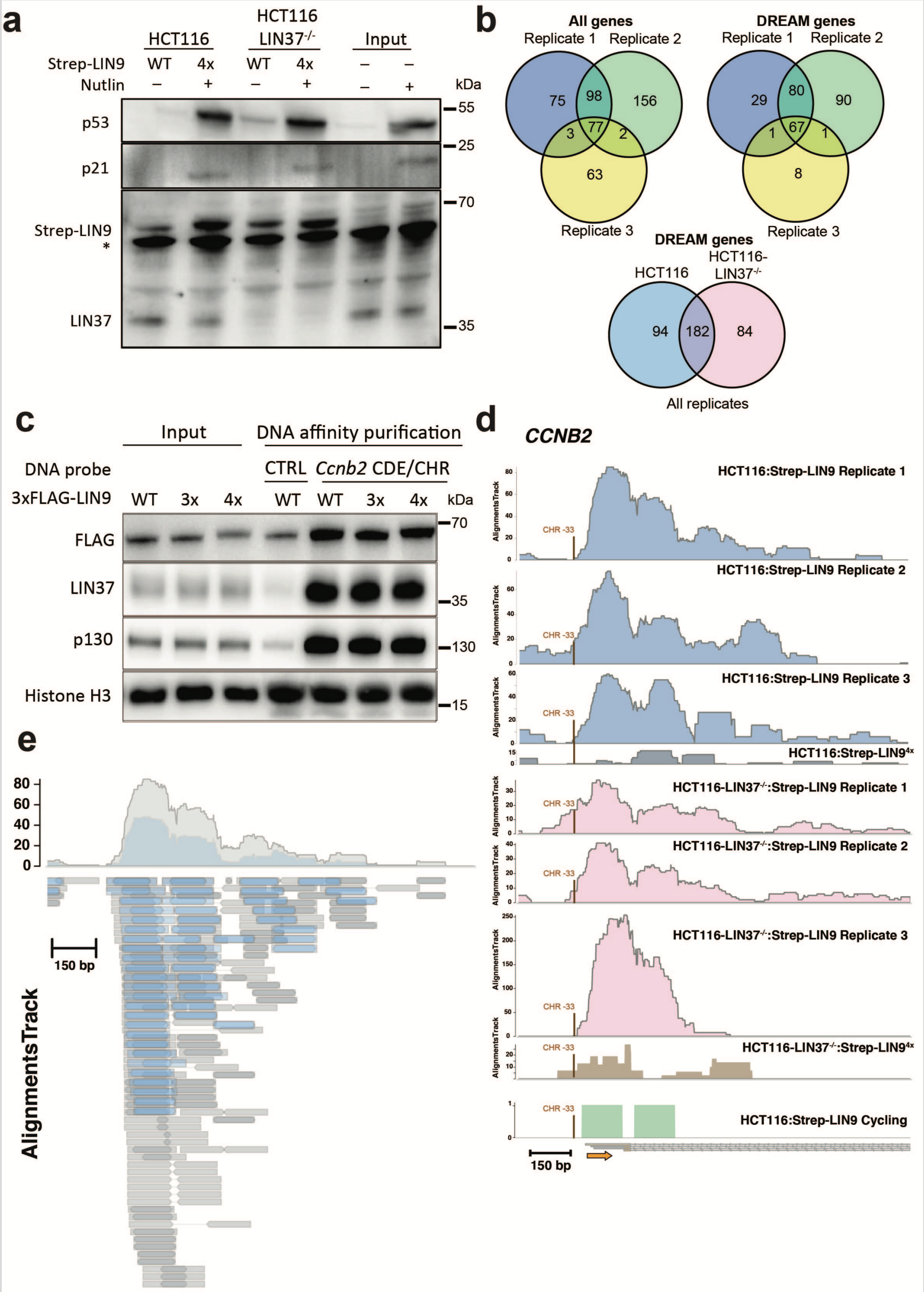

Supplementary Fig. 6: **Data supporting the MNase-ChIP experiment.** (**a**) Western blot demonstrates the expression of Strep-LIN9 and Strep-LIN9^{4x}, the absence of LIN37 in the HCT116-LIN37$^{-/-}$ cells, and the induction of p53 (antibody p53 (DO-1), RRID:AB_628082, Santa Cruz Biotechnology; dilution 1:1000) and the p53 target p21 (antibody p21 (F-5), RRID:AB_628073, Santa Cruz Biotechnology; dilution 1:500) upon Nutlin-3a treatment. * Indicates a nonspecific band in the blot. This experiment was performed with a biological replicate, and the results were similar. (**b**) Overlap of enriched genes between replicates of the MNase-ChIP experiment. The top two Venn diagrams show overlap of all genes (left) and DREAM genes (right) that were enriched >4.7-fold in the three replicate experiments in which Strep-LIN9 was expressed in HCT116 cells. The bottom Venn diagram shows overlap of genes that were enriched in experiments expressing Strep-LIN9 in HCT116 and HCT116-LIN37$^{-/-}$ cells. This comparison uses the total DREAM gene list compiled across all three replicates for each condition.

(**c**) LIN9 mutants that do not assemble LIN37 and/or RBAP48 still bind DNA containing the consensus CDE-CHR site. HCT116 cells were transfected with plasmids expressing 3xFLAG-tagged wild-type LIN9, LIN9^{3x}, and LIN9^{4x}. Cells were arrested with Nutlin-3a and MuvB complex components were purified. Purification was performed with a fragment of the pGL4.10 vector containing the mouse Ccnb2 CDE/CHR MuvB-binding site. Protein binding to a pGL4.10 fragment without this element was analyzed to control for background binding (CTRL). Binding of Flag-LIN9 and endogenous LIN37 and p130 was tested by western blotting. Histone H3 was probed as a control for DNA affinity purification. (**d**) Number of DNA sequence reads across the *CCNB2* gene track for all eight indicated experiments. The plots show DNA reads from the Strep-precipitated samples. The TSS (orange arrow) and position of the CHR element are shown. The number of reads from the Strep-LIN9^{4x} experiments are much lower and do not consistently show peaks corresponding to the +1 nucleosome. (**e**) DNA sequence reads from the Strep-LIN9:HCT116 precipitation are plotted for the *CCNB2* gene track as in Supplementary Fig. 6D. The alignment tracks of the sequence fragments are diagrammed below. In these tracks, explicitly sequenced DNA is shown as a block and inferred sequence from the paired end analysis is shown as a line. The grey tracks and coverage plot represent the full data as shown in Fig. 6d and Supplementary Fig. 6d. The blue tracks and coverage plot represent a subset of that data, in which the alignment tracks are filtered to include only sequence reads that are 130-200 base pairs, corresponding to mononucleosome-size fragments.

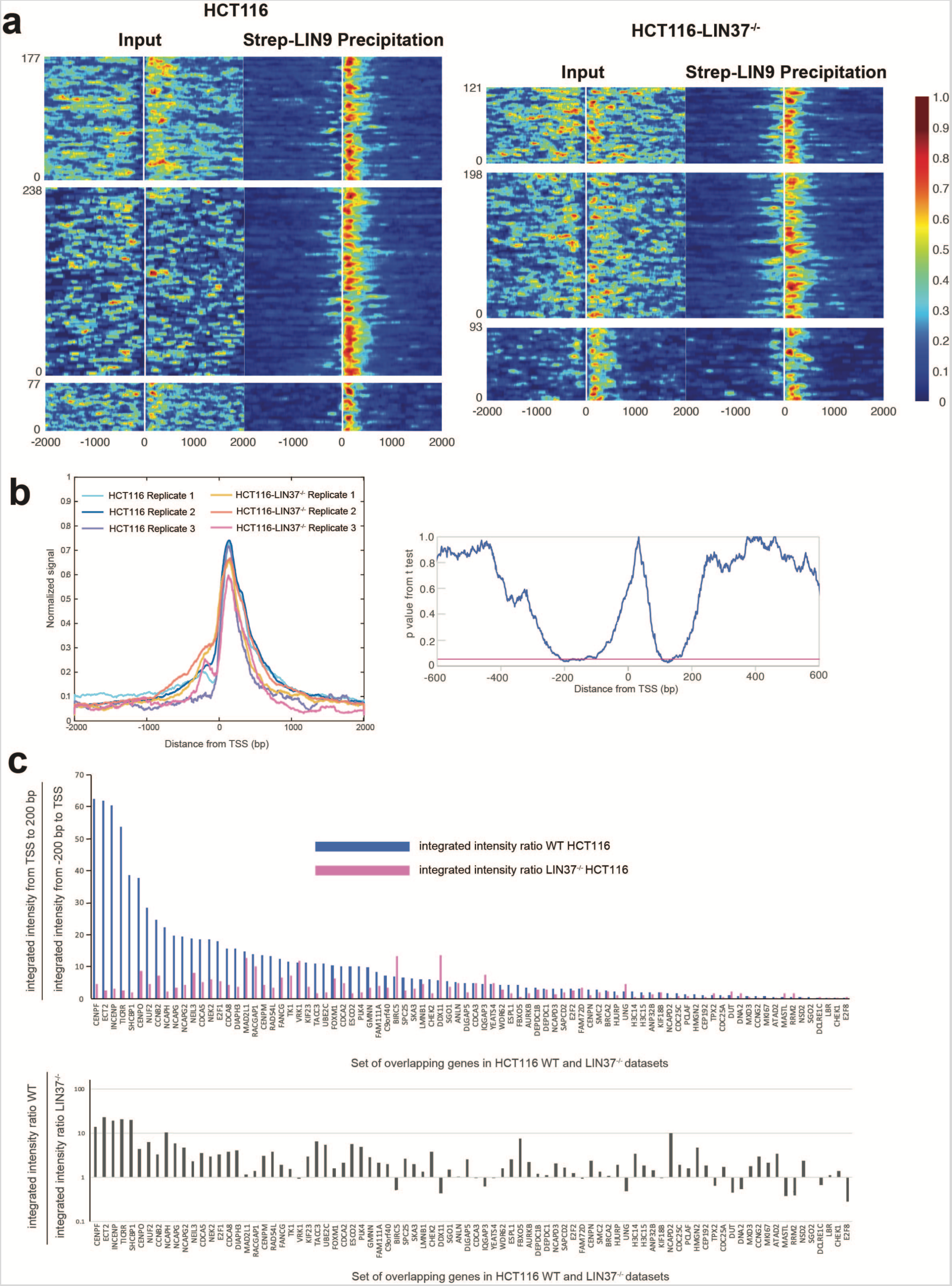

Supplementary Fig. 7: Comparison of MNase-ChIP replicate experiments in HCT116 and HCT116-LIN37$^{-/-}$ cells. Replicates 1 and 3 are biological replicates. Replicate 2 is a technical replicate of Replicate 1 but using a 5-fold lower MNase concentration. (**a**) As in Fig. 7B, heat maps for each experiment show the normalized DNA sequence read density across all DREAM genes that were detected with greater than 4.7-fold enrichment compared to a Strep-precipitation control. Replicates 1 to 3 are shown top to bottom in each cell type.

(**b**) As in Fig. 7B, overlay of the aggregated and normalized read signal density from the same set of genes as shown in the heat maps. The right graph shows results of a two-tailed student's t-test comparing the three replicate datasets from wild-type and the three replicate datasets from LIN37$^{-/-}$ HCT116 cells. The p-value comparing the normalized signal values in the two groups is reported for each base pair in the aggregated peaks. The pink line shows $p = 0.05$. (**c**) For every gene that is detected at greater than 4.7-fold enrichment in Replicate 1 of both the WT and LIN37$^{-/-}$ datasets, the ratio of the integrated normalized intensity of DNA reads 200 bp downstream to 200 bp upstream of the TSS is plotted (top). As seen in this plot and in panel B, in almost all cases the integrated intensity is greater downstream of the TSS (ratio greater than 1); however, the ratio of intensity is less for the experiment in the LIN37$^{-/-}$ cells, consistent with the conclusion that the nucleosome is less well positioned. To perform a statistical analysis, we calculated the ratio of these intensity ratios, comparing WT to LIN37$^{-/-}$ cells for each gene (plotted in bottom panel). The average ratio across the genes for this replicate (Replicate 1) was 3.5 and the 95% confidence interval, treating each gene as an individual experiment, is 2.5 to 4.4. We conclude that in LIN37$^{-/-}$ HCT116 cells, there is significantly less difference in nucleosome density between upstream and downstream of the TSS relative to WT HCT116 cells. We note that we obtained similar statistical significance when performing this analysis using data from the other two replicates of the experiment in WT and LIN37$^{-/-}$ HCT116 cells.
