# Supplementary material for: The MuvB complex binds and stabilizes nucleosomes downstream of the transcription start site of cell-cycle dependent genes
Source: Nat Commun. 2022 Jan 26;13:526. doi: 10.1038/s41467-022-28094-1 (PMC8792015; doi:10.1038/s41467-022-28094-1)
Supplement: Supplementary file 3 — Reporting Summary [file 41467_2022_28094_MOESM3_ESM.pdf]

nature portfolio

Corresponding author(s): Seth M. Rubin

Last updated by author(s): 2021-Dec-26

Reporting Summary

Nature Portfolio wishes to improve the reproducibility of the work that we publish. This form provides structure for consistency and transparency in reporting. For further information on Nature Portfolio policies, see our Editorial Policies and the Editorial Policy Checklist.

Statistics

For all statistical analyses, confirm that the following items are present in the figure legend, table legend, main text, or Methods section.

n/a	Confirmed	
☐	☒	The exact sample size (*n*) for each experimental group/condition, given as a discrete number and unit of measurement
☐	☒	A statement on whether measurements were taken from distinct samples or whether the same sample was measured repeatedly
☐	☒	The statistical test(s) used AND whether they are one- or two-sided<br>*Only common tests should be described solely by name; describe more complex techniques in the Methods section.*
☒	☐	A description of all covariates tested
☐	☒	A description of any assumptions or corrections, such as tests of normality and adjustment for multiple comparisons
☐	☒	A full description of the statistical parameters including central tendency (e.g. means) or other basic estimates (e.g. regression coefficient) AND variation (e.g. standard deviation) or associated estimates of uncertainty (e.g. confidence intervals)
☐	☒	For null hypothesis testing, the test statistic (e.g. *F*, *t*, *r*) with confidence intervals, effect sizes, degrees of freedom and *P* value noted<br>*Give P values as exact values whenever suitable.*
☒	☐	For Bayesian analysis, information on the choice of priors and Markov chain Monte Carlo settings
☒	☐	For hierarchical and complex designs, identification of the appropriate level for tests and full reporting of outcomes
☒	☐	Estimates of effect sizes (e.g. Cohen's *d*, Pearson's *r*), indicating how they were calculated

Our web collection on statistics for biologists contains articles on many of the points above.

Software and code

Policy information about availability of computer code

Data collection	JBluIce unknown version
Data analysis	ImageJ 1.52 , PRISM 8.2.1, Coot 0..8.9.2, CCP4 7.0.076, mosflm 7.2.2, pymol 1.7.6.0, bwa-mem 0.7.17, macs2 2.2.7.1, Panther 16.0, REVIGO unknown version (web tool run May 2021), bedtools 2.26.0, samtools 1.5, Nuctools unknown version, R 4.0.3, and MATLAB R2020a

For manuscripts utilizing custom algorithms or software that are central to the research but not yet described in published literature, software must be made available to editors and reviewers. We strongly encourage code deposition in a community repository (e.g. GitHub). See the Nature Portfolio guidelines for submitting code & software for further information.

Data

Policy information about availability of data

All manuscripts must include a data availability statement. This statement should provide the following information, where applicable:

- Accession codes, unique identifiers, or web links for publicly available datasets
- A description of any restrictions on data availability
- For clinical datasets or third party data, please ensure that the statement adheres to our policy

The following statement has been added to the manuscript:
X-ray diffraction data and model coordinates for the MuvBN structure in this study have been deposited in the Protein Data Bank (RCSB.org) under accession code 7N40.Previously determined structures that were used for comparison are also available in the Protein Data Bank under accession codes 2YBA, 3CFV, 3GFC, 2M0O, 4A4E, 2XU7, 4R7A, 4PC0, 2YB8, 5FXY, 5WAI. MNase-ChIP data have been deposited in the NCBI GEO database (https://www.ncbi.nlm.nih.gov/geo/) under accession code GSE189435. Source data are provided with this paper.

Field-specific reporting

Please select the one below that is the best fit for your research. If you are not sure, read the appropriate sections before making your selection.

☒ Life sciences ☐ Behavioural & social sciences ☐ Ecological, evolutionary & environmental sciences

For a reference copy of the document with all sections, see nature.com/documents/nr-reporting-summary-flat.pdf

Life sciences study design

All studies must disclose on these points even when the disclosure is negative.

Sample size	No sample size calculation was performed. In the experiment detailed in Figure 5, an initial guess was made by choosing at least 100 molecules per condition with the assumption that this would generate good Poisson statistics. Our statistical analysis indeed showed significant differences in the experimental and control conditions.
Data exclusions	None
Replication	Protein-coprecipitation and biophysical assays were performed in triplicate. MNase-ChIP experiments were performed with one technical and one biological replicate.
Randomization	Not applicable. The experimental protocols used in this study do not present any obvious need for randomization.
Blinding	For the experiment in Figure 5, separate researchers performed different aspects of the data analysis such to minimize bias. Molecules on the EM grid were chosen for analysis without knowing sample identity. Other experiments were performed by a single researcher without blinding.

Reporting for specific materials, systems and methods

We require information from authors about some types of materials, experimental systems and methods used in many studies. Here, indicate whether each material, system or method listed is relevant to your study. If you are not sure if a list item applies to your research, read the appropriate section before selecting a response.

Materials & experimental systems

n/a	Involved in the study	
☐	☒	Antibodies
☐	☒	Eukaryotic cell lines
☒	☐	Palaeontology and archaeology
☒	☐	Animals and other organisms
☒	☐	Human research participants
☒	☐	Clinical data
☒	☐	Dual use research of concern

Methods

n/a	Involved in the study	
☐	☒	ChIP-seq
☒	☐	Flow cytometry
☒	☐	MRI-based neuroimaging

Antibodies

Antibodies used	FLAG-HRP (RRID:AB_2017593, Santa Cruz Biotechnology), p130/RBL2 (D9T7M) (RRID:AB_2798274, Cell Signaling), LIN54 A303-799A (RRID:AB_11218173, Bethyl Laboratories), LIN9 ab62329 (RRID:AB_1269309, Abcam), RBBP4 A301-206A (RRID:AB_890631, Bethyl Laboratories), LIN37-T3 (custom-made at Pineda Antikörper-Service, Berlin, Germany), FLAG-M2 (RRID:AB_262044, Sigma-Aldrich), Histone H3 (RRID:AB_331563, Cell Signaling Technology)
Validation	Commercially available antibodies were validated by manufacturer for immunoprecipitation and immunoblotting. See manufacturers website. Validation by gene knockout of the custom made LIN37-T3 antibody is reported in PMID: 28920576

Eukaryotic cell lines

Policy information about cell lines

Cell line source(s)	HCT-116 cells were purchased from the ATCC. Sf9 cells were purchased from Expression Systems.
Authentication	Authentication performed by ATCC and Expression Systems.
Mycoplasma contamination	HCT116 cells tested negative for mycoplasma. Sf9 cells not tested by us.
Commonly misidentified lines (See ICLAC register)	None

ChIP-seq

Data deposition

☒ Confirm that both raw and final processed data have been deposited in a public database such as GEO.

☒ Confirm that you have deposited or provided access to graph files (e.g. BED files) for the called peaks.

Data access links<br>*May remain private before publication.*	https://www.ncbi.nlm.nih.gov/geo/query/acc.cgi?acc=GSE189435
Files in database submission	Raw paired end fastq files, macs2 raw .broadpeak files, and annotated peak files in .txt format have been deposited.
Genome browser session<br>(e.g. UCSC)	Did not use -- TDF and bam files for browsing are provided in the link

Methodology

Replicates	One technical and one biological replicate; comparison of replicates shown in Figure 6 supplement
Sequencing depth	We targeted 20-25 million reads per condition on the 150bp paired-end sequencer (~about 1-2x coverage per condition).
Antibodies	We used anti-strep coated magnetic beads: https://www.iba-lifesciences.com/strep-tactin-magnetic-microbeads/6-5510-050
Peak calling parameters	macs2(version: macs2 2.2.7.1) peak calling. Parameters: callpeak -t WT_1M_LIN9_IP.bam -c WT_1M_mock_IP.bam --broad -g hs --broad-cutoff 0.1 -f BAMPE -n 1MStrepLin9kJ s
Data quality	We report number of peaks greater than 4.7-fold enrichment in figure 6C and Supp Figure 6C showing the comparison across all replicates.
Software	bwa-mem 0.7.17, MACS2 2.2.7.1, Nuctools (unknown version), samtools 1.5, bedtools 2.26.0 J
